# The complete mitochondrial genome of *Anthrenus museorum* (Coleoptera: Bostrichiformia: Dermestidae) from China

**DOI:** 10.1080/23802359.2023.2187655

**Published:** 2023-03-16

**Authors:** Yangyang Liu, Yaoyao Dong, Zhenglong Ye, Siyong Wu, Guoyong Li

**Affiliations:** Guizhou Provincial Key Laboratory for Rare Animal and Economic Insects of Mountainous Region, College of Biological and Environmental Engineering, Guiyang University, Guiyang, P. R. China

**Keywords:** Dermestid beetle, pest, mitogenomes, phylogenetic analysis

## Abstract

Dermestid beetles (Coleoptera: Bostrichiformia: Dermestidae) are important pests of various storage products and pose a potential threat to international trade. In this study, the whole mitogenome of *Anthrenus museorum* was first sequenced and annotated and was found to have the same gene order observed in known dermestid beetles. It comprised 13 protein-coding genes (PCGs), 22 transfer RNAs, 2 ribosomal RNAs and a control region. The typical ATN start codon was observed in all PCGs, except for *ND3* (TTG), and all 13 PCGs showed three types of stop codons (TAA, TAG, and T-). Phylogenetic analysis based on the PCGs indicated that the relationships within Bostrichiformia were reconstructed, with the exception of one early emerging species of Bostrichidae that actually makes the group polyphyletic, as (Dermestidae + (Bostrichidae + Anobiidae)). Moreover, it revealed a close relationship between *A. museorum* and *A. verbasci* using maximum likelihood and Bayesian inference analysis.

## 1. Introduction

*Anthrenus museorum* (Linnaeus, 1761), belonging to the family Dermestidae (Vikberg 1999, Kadej et al. 2013), is a pest with strong adaptability and a wide range of harmful effects in various stores, such as animal carcasses, feathers, aquatic products, skin, and medicinal materials (Rajendran and Parveen 2005). It is also one of the primary hazardous pests in museum exhibition halls and specimen warehouses, particularly insect specimen warehouses. *A. museorum* is native to Europe and is globally dispersed through commercial logistics transmission (Bergh et al. 2003). However, dermestid beetles that are intercepted during quarantine at Chinese ports are similar in morphology and biological habits (Holloway and Pinninger 2020) and cannot be distinguished by morphology alone. Recently, molecular approaches have been employed for dermestid beetle identification, using a particular mitochondrial gene (Cytochrome oxidase 1 [*COX1*]). Currently, there are scarce molecular data regarding Dermestidae and Bostrichiformia insects in GenBank, with incomplete mitochondrial gene sequences (e.g. *12S*, *16S* genes). However, extensive studies have revealed that the species cannot be accurately identified using molecular fragments alone and that new genome-level molecular markers are constantly being developed and widely used in several non-model species (Brown et al. 1979; Ballard and Whitlock 2004). This is the first study to perform sequencing of the complete mitogenome of *A. museorum*, which is a useful tool for the accurate identification of this family and helps in the enrichment of the mitochondrial genome database.

## 2. Materials

**Figure 1.**
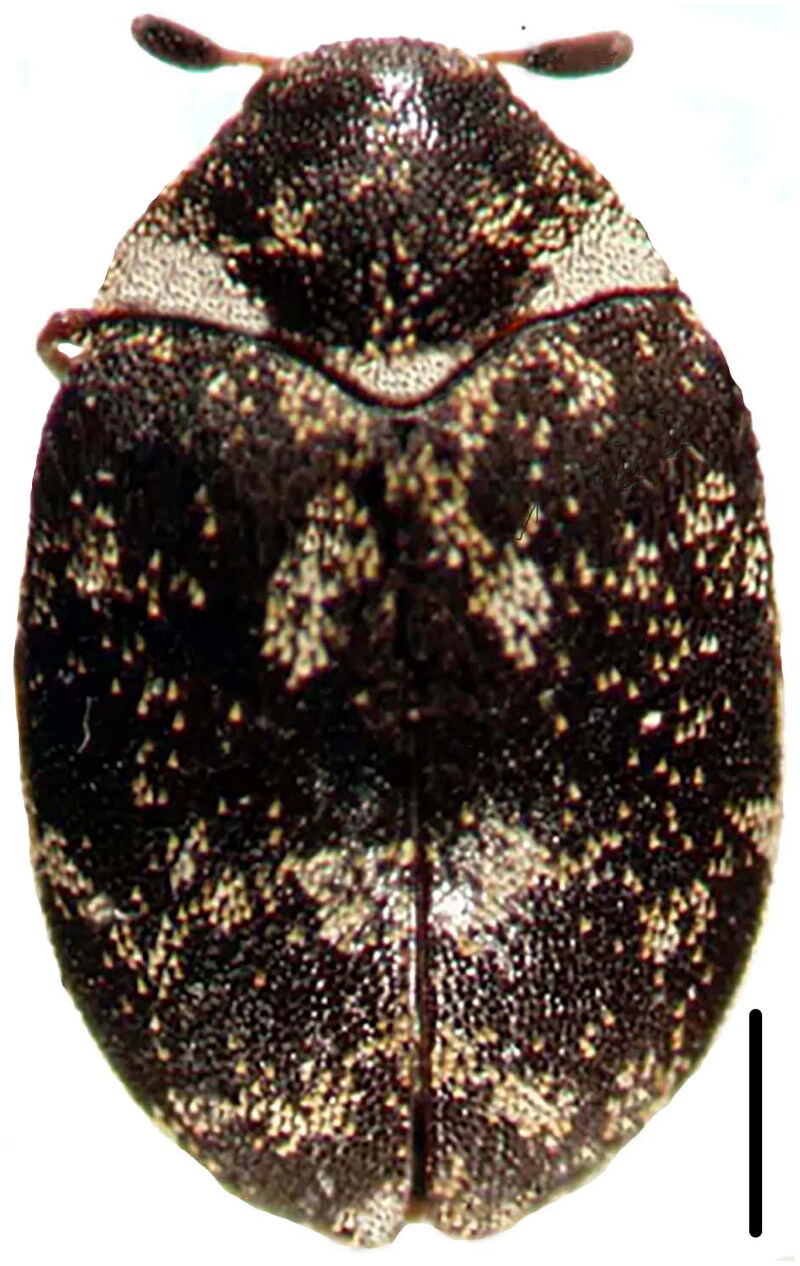
The specimen map of *A. museorum*. (Scale = 0.5 mm. This is a species reference image by Yangyang Liu, the first author of this article).

*A. museorum* samples were collected from the thorax of *Trypoxylus dichotomus* (Linnaeus, 1771) from Huaxi District, Guiyang, Guizhou Province, China (26°25′N, 106°38′E), the samples were deposited in the animal laboratory of the Guiyang University (Yangyang Liu) under the voucher number GYU-20220220-002 (Figure 1). The insects were caught and immediately soaked in alcohol, which resulted in sudden death and allowed DNA extraction immediately after capture. As the insects were arthropods, no ethical considerations were needed for this research.

## 3. Methods

### 3.1. DNA extraction

DNA was extracted from 10 individuals of *A. museorum* samples using TIANGEN Genomic DNA Extraction Kit (DP304) (TIANGEN Co., Beijing, China), according to the manufacturer’s protocol. The concentration and purity of total DNA were assessed using a nucleic acid analyzer (NanoDrop 2000, Thermo Fisher Scientific, USA).

### 3.2. Sequencing, assembly, and annotation

After quality assessment, the library was constructed from a pooled DNA sample and sequencing was performed by the Berry Genomics Biotechnology company (Beijing, China). The genomic DNA samples were enzymatically disrupted to fragments of 350 bp in length using TruSeq Nano DNA HT Sample Preparation Kit (Illumina, USA). Next, they were sequenced using Illumina NovaSeq 6000 with 150-bp paired-end reads, and the raw data were processed using NGS QC toolkit (Patel and Jain 2012) (Supplementary Material, Figure S1). Clean data were de novo assembled using Mitoz v. 2.3 software (Meng et al. 2019) (Supplementary Material, Figure S2 and S3). Geneious R10 (settings: min overlap identity = 95%, min overlap = 50 bp, max gap size = 20 bp, and max gaps/read = 5%) and MITOS (settings: reference = RefSeq 63 Metazoa, genetic code = 5 invertebrate) were used to annotate assembled mitogenome (Kearse et al. 2012; Bernt et al. 2013), and corrected by downloading annotation results from mitochondrial genomes of closely related species in GenBank (Altschul et al. 1990). The annotate complete mitogenome sequence and raw data were submitted to GenBank and Sequence Read Archive, respectively. The mitogenome map of *A. museorum* was generated by Geneious R10 (Kearse et al. 2012).

**Figure 2.**
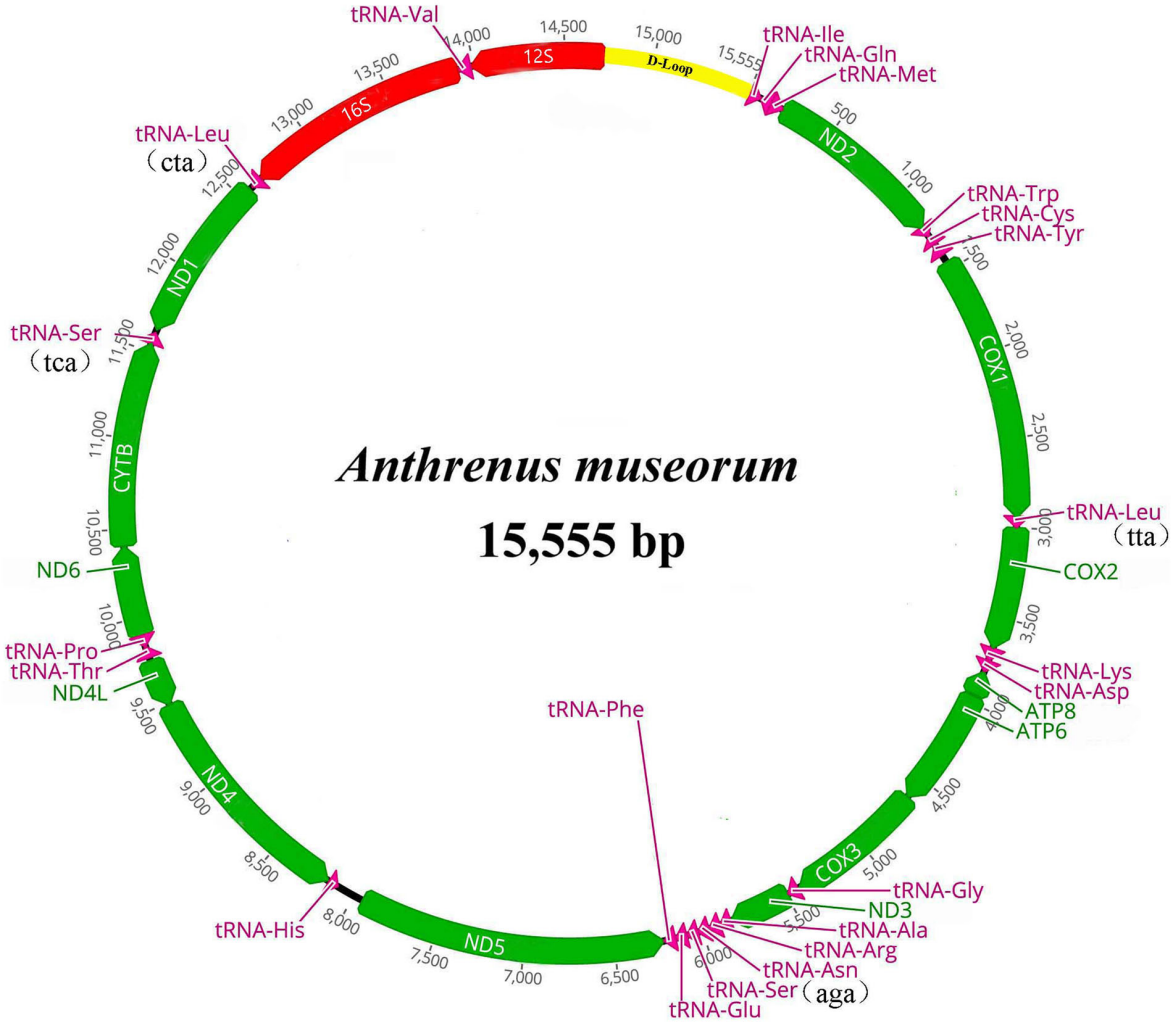
Circular map of the mitogenome of *A. museorum* mitogenomes. Genes indicated by different colors. Protein-coding genes, transfer RNA, ribosomal RNA, and control region are represented by yellow or green, purple, red and blue, respectively.

**Figure 3.**
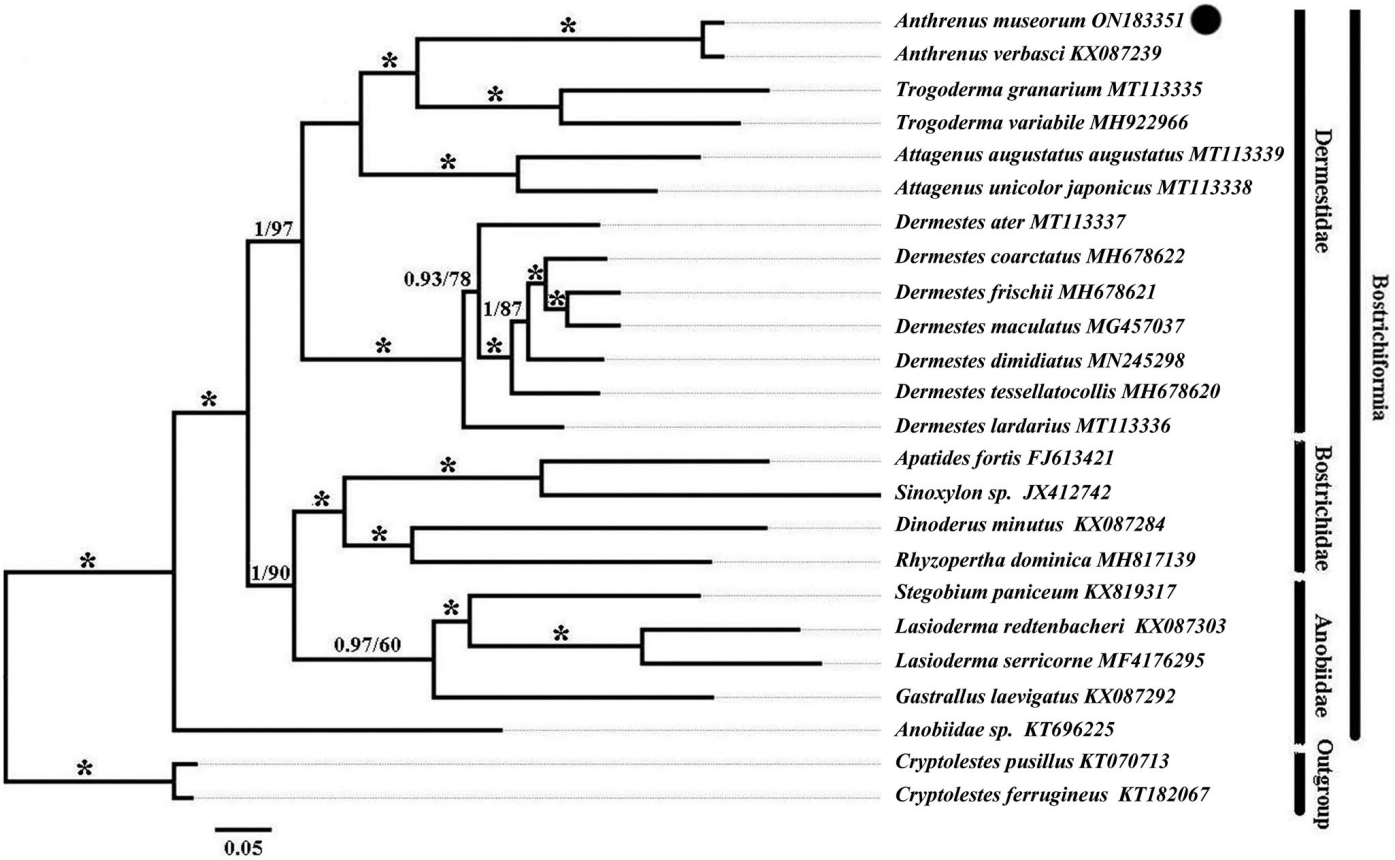
Phylogenetic relationships were constructed using the Bayesian inference (BI) and maximum likelihood (ML) analysis methods. Numbers on branches indicate BI posterior probability (left) and ML bootstrap support (right). Asterisk indicates 1/100. *A. museorum* collected from the Guizhou Province, China, is marked with a black dot.

### 3.3. Phylogenetic analysis

Phylogenetic analyses were conducted based on the mitogenomes of 24 species of Coleoptera, including all 22 available genomes from Bostrichiformia, and two species of Cucujiformia (*Cryptolestes ferrugineus* *and* *C. pusillus*), which served as outgroups (Li et al. 2016; Zeng et al. 2020; Wang et al. 2020; Wu et al. 2020) (Table 1). All sequences were aligned using MEGA X (Kumar et al. 2018) with the default settings, and the resulting alignments from the sequences of 13 protein-coding genes (PCGs) were concatenated into sets of combined sequences using SequenceMatrix 1.8 (Vaidya et al. 2011). The optimal partitioning scheme and models were obtained by using PartitionFinder2 (algorithm = greedy, min-subset-size = 1000) (Supplementary Material, Table S1) (Lanfear et al. 2017). Phylogenetic trees were constructed using the Bayesian inference (BI) and maximum likelihood (ML) methods *via* MrBayes 3.2 (mcmcp ngen = 10,000,000, relburnin = yes, samplefreq = 1000, printfreq = 1000, nchains = 4, savebrlens = yes) (Ronquist et al. 2012) and IQ-TREE 1.6.2 (1000 bootstrap iterations) (Nguyen et al. 2015), respectively.

**Table 1.** List of the mitogenomic records from 24 Coleoptera species analyzed in this study and their GenBank accession numbers and publication source.

Infraorder	Family	Species	Accession no.	Articles cited
Bostrichiformia	Dermestidae	*Trogoderma granarium*	MT113335	Zeng et al. 2020
		*Trogoderma variabile*	MH922966	Zeng et al. 2020
		*Dermestes dimidiatus*	MN245298	Wang et al. 2020
		*Dermestes ater*	MT113337	Zeng et al. 2020
		*Dermestes lardarius*	MT113336	Zeng et al. 2020
		*Dermestes maculatus*	MG457037	Zeng et al. 2020
		*Dermestes coarctatus*	MH678622	Zeng et al. 2020
		*Dermestes frischii*	MH678621	Wang et al. 2020
		*Dermestes tessellatocollis*	MH678620	Wang et al. 2020
		*Attagenus augustatus augustatus*	MT113339	Zeng et al. 2020
		*Attagenus unicolor japonicus*	MT113338	Zeng et al. 2020
		*Anthrenus verbasci*	KX087239	Zeng et al. 2020
		*Anthrenus museorum*	ON183351	This study
	Bostrichidae	*Rhyzopertha dominica*	MH817139	Wang et al. 2020
		*Dinoderus minutus*	KX087284	Wang et al. 2020
		*Sinoxylon* sp.	JX412742	Wang et al. 2020
		*Apatides fortis*	FJ613421	Wu et al. 2020
	Anobiidae	*Lasioderma serricorne*	MF417629	Wang et al. 2020
		*Lasioderma redtenbacheri*	KX087303	Wang et al. 2020
		*Stegobium paniceum*	KX819317	Wang et al. 2020
		*Gastrallus laevigatus*	KX087292	Wang et al. 2020
		*Anobiidae* sp.	KT696225	Wu et al. 2020
Cucujiformia (Outgroup)	Laemophloeidae	*Cryptolestes ferrugineus*	KT182067	Zeng et al. 2020
		*Cryptolestes pusillus*	KT070713	Li et al. 2016

## 4. Results

### 4.1. Mitogenome organization and base composition

The raw data size was 11.89Gbp, with Q20 of >96.57%, and the clean data size was 11.77 Gbp, with Q20 of >96.66% (Supplementary Material, Figure S1 and S2). The mitogenome of *A. museorum* was 15,555 bp long, and the sequence was submitted to the GenBank database with accession no. ON183351. Circular DNA comprised 42.3% A, 31.1% T, 16.9% C, and 9.7% G, with A + T-bias (73.4%). The whole genome included 13 PCGs, 22 transfer RNAs (tRNAs), 2 ribosomal RNAs (rRNAs), and 1 noncoding control region (D-loop). The N-strand -contained 13 genes, including 5 PCGs (*ATP8*, *ND1*, *ND4*, *ND4L*, and *ND5*), 8 tRNAs (tRNA-*Gln*, tRNA-*Cys*, tRNA-*Tyr*, tRNA-*Phe*, tRNA-*His*, tRNA-*Pro*, tRNA-*Leu* (cta), and tRNA*-Val*), and 2 rRNAs (*16S* and *12S*); The J-strand- contained 8 PCGs (*COX1*, *COX2*, *COX3*, *CYTB*, *ND2*, *ND3*, *ND6*, and *ATP6*), and remaining 14 tRNAs (Figure 2). The typical ATN start codon was observed in all PCGs, except for *ND3* (TTG), and all 13 PCGs showed three types of stop codons (TAA, TAG, and T-).

### 4.2. Phylogenetic analysis

In this study, phylogenetic analyses were based on the nucleotide sequences of the 13 PCGs obtained from the mitogenomes of 22 species of Bostrichiformia and the outgroups (*C. ferrugineus and C. pusillus*), using the BI and ML methods (Figure 3). The phylogenetic topology results of these two methods were identical, and the phylogenetic relationships were as follows: (Anobiidae + ((Bostrichidae + Anobiidae) + Dermestidae)). Apart from the position of sequence Anobiidae sp. KT696225, which emerges as basal to all other Bostrchiformia thus making the family Anobiidae polyphyletic, relationships at the family level are recovered as (Dermestidae + (Bostrichidae + Anobiidae)). The results of both phylogenetic trees revealed that *A. museorum* is more closely related to *A. verbasci*, and on this branch, the posterior probability of the Bayesian phylogenetic tree on this branch and the bootstrap value of the ML phylogenetic tree is 1/100, respectively.

## 5. Discussion and conclusion

The mitogenome of *A. museorum.* was 15,555 bp long, and the A + T bias (73.4%) indicated the overall nucleotide composition of the genome, which was 69.5–84.9%, similar to that of other arthropods with significant variability in A + T content (Dotson and Beard 2001). The gene order was identical to that of other known mitogenomes of dermestid beetles (Zeng et al. 2020, Wang et al. 2020). In addition, the *ND3* (TTG) did not contain the typical ATN start codon, which is observed in the other 12 PCGs. The results showed that the topologies of the phylogenetic trees constructed using the two methods were similar, which was consistent with the traditional morphological classification (Holloway and Pinninger 2020). The results of both phylogenetic trees showed that the mitochondrial DNA of *A. museorum* was closely related to that of *A. verbasci*. Although only a few species of Dermestidae were included in this study, the results suggest that the mitogenome is useful as a genetic marker and is a feasible method for the systematic classification of Dermestidae. Further information regarding the mitogenome of Dermestidae insects is required in the future.

## Supplementary Material

Supplemental Material

Supplemental Material

Supplemental Material

Supplemental Material

## Data Availability

The genome sequence data that support the findings of this study are openly available in GenBank of NCBI at [https://www.ncbi.nlm.nih.gov/] under the accession no. ON183351. The associated BioProject, SRA, and Bio-Sample numbers are PRJNA824543, SRS12541364, and SAMN27407755, and all accession numbers are activated.
