# Supplementary material for: The complete mitochondrial genome of Anthrenus museorum (Coleoptera: Bostrichiformia: Dermestidae) from China
Source: Mitochondrial DNA B Resour. 2023 Mar 16;8(3):405–9. doi: 10.1080/23802359.2023.2187655 (PMC10324981; doi:10.1080/23802359.2023.2187655)
Supplement: Supplemental Material [file TMDN_A_2187655_SM6033.doc]

**Table S1 Best partitioning scheme and models for 13PCGs data matrix selected by PartitionFinder2.**

| **Data matrix** | **Method** | **Subset Partitions** | **Best Model** |
| --- | --- | --- | --- |
| 13PCGs | Bayesian Inference (BI) - MrBayes | *ATP6* | GTR+I+G |
|  |  | *ATP8 ND6* | GTR+I+G |
|  |  | *COX1* | GTR+I+G |
|  |  | *COX2* | GTR+I+G |
|  |  | *COX3 CYTB* | GTR+I+G |
|  |  | *ND1* | GTR+I+G |
|  |  | *ND2* | GTR+I+G |
|  |  | *ND3* | GTR+I+G |
|  |  | *ND4 ND4L* | GTR+I+G |
|  |  | *ND5* | GTR+I+G |
|  | Maximum Likelihood (ML) - IQ | *ATP6 COX3 CYTB* | GTR+F+I+G4 |
|  |  | *ATP8* | TN+F+I+G4 |
|  |  | *COX1 COX2* | GTR+F+I+G4 |
|  |  | *ND1* | TIM+F+I+G4 |
|  |  | *ND2* | GTR+F+I+G4 |
|  |  | *ND3 ND6* | GTR+F+I+G4 |
|  |  | *ND4L* | TIM+F+I+G4 |
|  |  | *ND4 ND5* | GTR+F+I+G4 |
