# Supplementary figures and images for: The complete mitochondrial genome of Anthrenus museorum (Coleoptera: Bostrichiformia: Dermestidae) from China
Source: Mitochondrial DNA B Resour. 2023 Mar 16;8(3):405–9. doi: 10.1080/23802359.2023.2187655 (PMC10324981; doi:10.1080/23802359.2023.2187655)

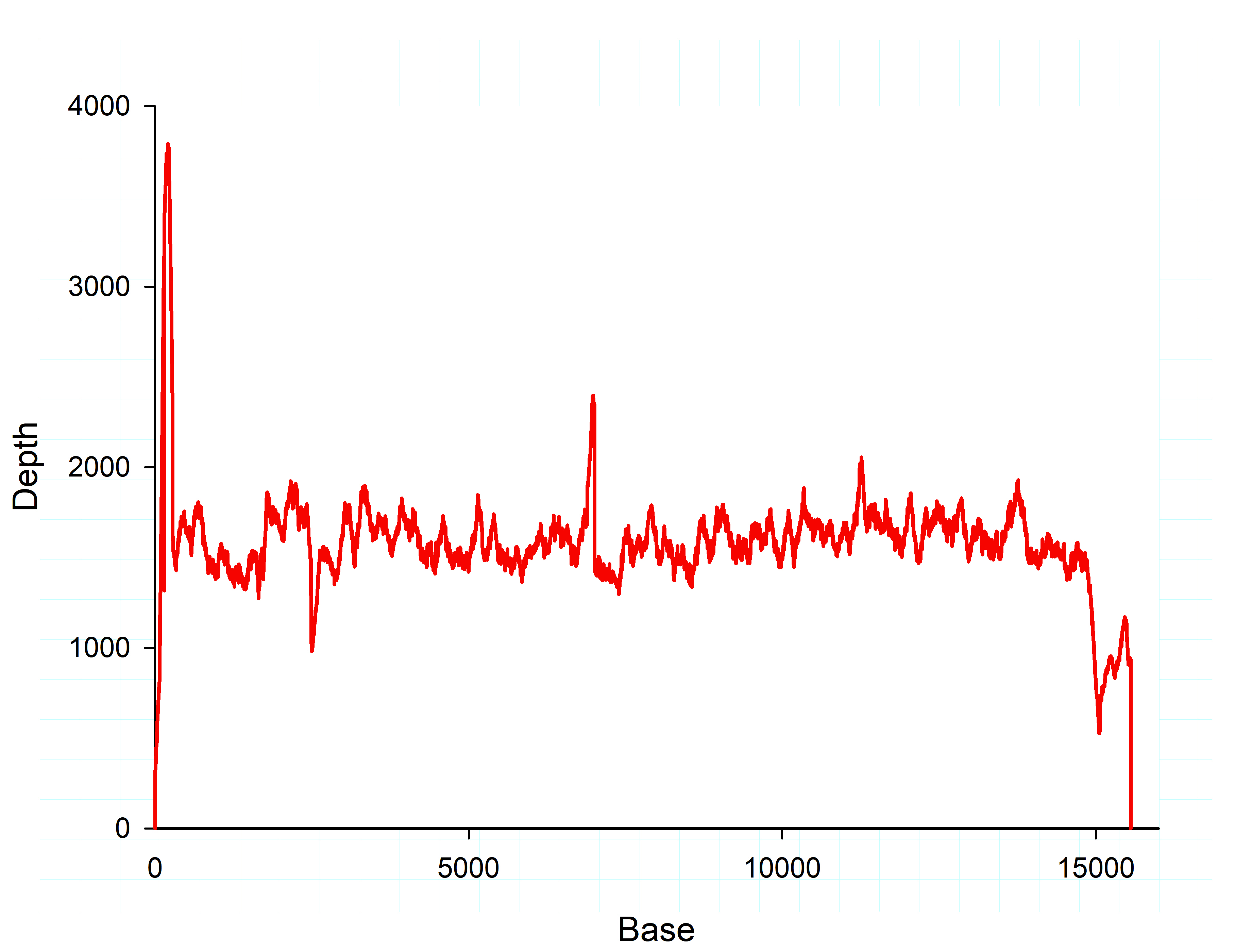

Supplement: Supplemental Material [file TMDN_A_2187655_SM6032.jpg]

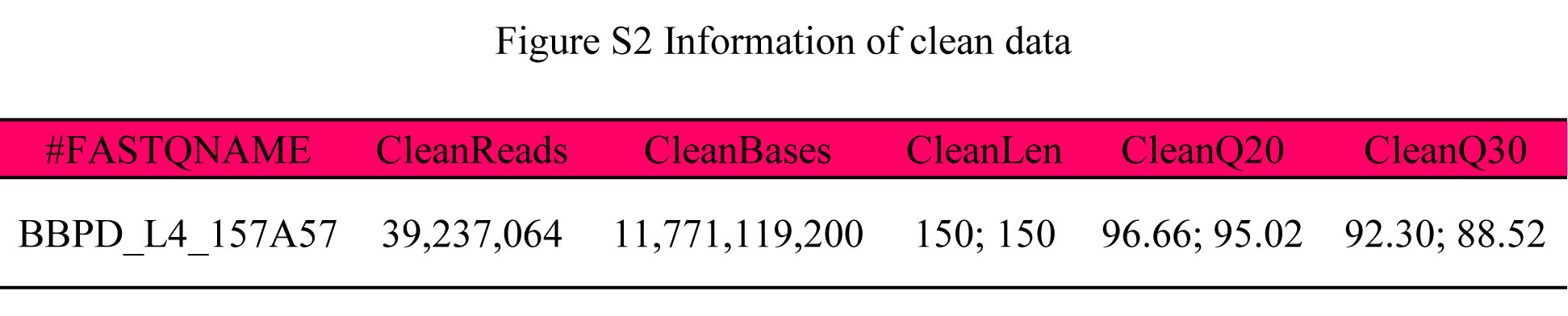

Supplement: Supplemental Material [file TMDN_A_2187655_SM6027.jpg]

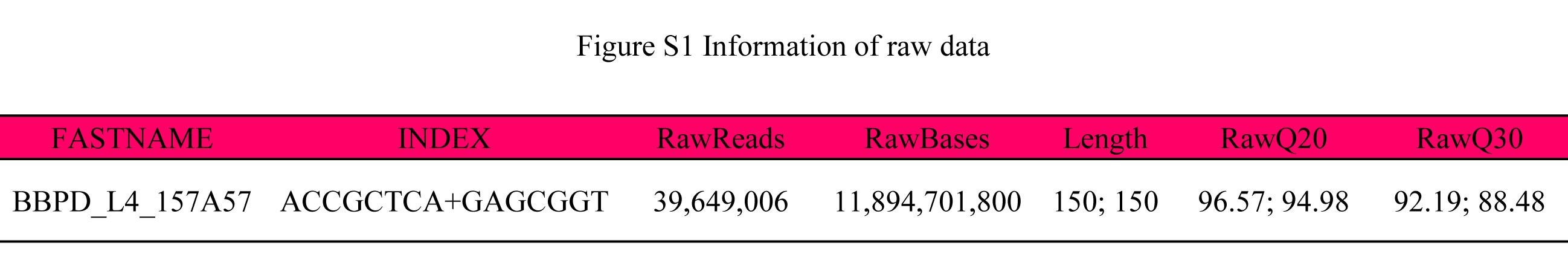

Supplement: Supplemental Material [file TMDN_A_2187655_SM6026.jpg]
